# Plasma-based fast-gelling biohybrid gels for biomedical applications

**DOI:** 10.1038/s41598-019-47366-3

**Published:** 2019-07-26

**Authors:** Amrita Pal, Kunal Tripathi, Chandrashekhar Pathak, Brent L. Vernon

**Affiliations:** Arizona State University, Tempe, AZ 85287 USA

**Keywords:** Drug delivery, Gels and hydrogels

## Abstract

Blood based biomaterials are widely researched and used in different biomedical applications including cell therapy, drug delivery, sealants etc. due to their biocompatibility and biodegradability. Blood derived gels are successfully used in clinical studies due to the presence of fibrinogen and several platelet growth factors. In spite of their wide applications, it is challenging to use blood-based biomaterials due to their low mechanical stability, poor adhesive property and contamination risk. In this study, we used porcine plasma to form gel in presence of biodegradable synthetic crosslinkers. Mechanical strength of this plasma gel could be tailored by altering the amount of crosslinkers for any desired biomedical applications. These plasma gels, formed by the synthetic crosslinkers, were utilized as a drug delivery platform for wound healing due to their low cytotoxicity. A model drug release study with these plasma gels indicated slow and sustained release of the drugs.

## Introduction

The development of *in situ* forming biomaterials has received considerable attention over the last few years^1–3^. These biomaterials have been widely used in soft tissue engineering^4,5^, drug delivery systems^2,6–10^ as well as surgical sealants^11–17^ as they provide a compatible microenvironment for drugs and cells. Aqueous solutions of these biomaterials, containing drugs or cells, form *in situ* gels triggered by one or a combination of several factors like temperature, pH change, presence of ions, solvent exchange and ultra violet irradiation^1^. During this sol-gel transition, the drugs or cells are entrapped in the gel without damage, and then the drugs can slowly be released, or cells can grow in a controlled fashion.

Biomaterials, derived from human blood (e.g. fibrin sealant/glue, platelet gel, and platelet fibrin glue), have also been studied extensively for their critical advantages including biocompatibility and biodegradability by enzymes, present *in situ*. They are used in clinical practice due to high content in fibrinogen or multiple platelet growth factors^18^. Several fibrin-based, *in situ* gels (injectable or implantable) have been developed for cell carrier, wound healing, surgical sealant and tissue or cartilage engineering applications^5,19–24^. Further, fibrin sealants have been used for more than 30 years as surgical hemostatic and sealing agents^25^. In some recent studies, fibrin sealants have been used as a platform for controlled drug release as well as a substrate for cellular growth and tissue engineering^25–27^. Platelet gels (PG) are a recently developed biomaterial which is prepared by combining platelet-rich blood/plasma, with calcified thrombin and has been used in bone reconstruction, tissue engineering, regenerative medicine, orthopedic and cosmetic surgery^25,28,29^. Promising therapeutic efficacy of platelet gels have been recently observed in the in vivo myocardial infraction models^30,31^. Platelet fibrin glue is another recently developed blood-derived biomaterial, which is prepared by combining PG with fibrinogen. This biomaterial, containing platelet growth factors and having moderate tensile strength, has wide application in tissue reconstruction, cancer surgery, liver transplantation, drug treatment, and orthopedic and reconstructive bone surgery^25,32^.

In spite of the various advantages of synthetic biomaterials (cyanoacrylates, PEG etc.) in wound-closure and tissue engineering, they also have several limitations including cytotoxicity, chronic inflammation, low adherence to the wet tissues and, in some cases, uncontrollable swelling as well as long curing time are some of the limitations associated with synthetic-based sealants^33,34^. On the other hand, a number of natural semi-synthetic biomaterial including fibrin, gelatin, albumin, chitosan, dextran etc. has been developed which exhibited great potential to act as surgical sealants. Although a few of them are clinically safe and can be used without any issue, the rests have some limitations including low mechanical stability poor adhesion strength and risk of bacterial and viral contamination, etc.^33,35^. To increase the mechanical stability, gelatin and albumin are mixed with toxic agents including formaldehyde and glutaraldehyde which causes inflammation in the wound^34,36^. Although, in a recent study, better mechanical property of human plasma gel was observed^37^ but the use of transglutaminase as the gelling reagent might make the gel expensive. Further, these surgical sealants are also relatively expensive compared with traditional surgical techniques. To address these many issues, an alternative approach to these biomaterials has been developed where *in situ* biogels, using blood/plasma, could be formed directly and it can be used either as a vehicle for controlled drug release in the wound or as a surgical sealant or both at the same time.

In our study, we have developed a new blood-based biomaterial, forming a hybrid clot by using synthetic crosslinkers which are able to gel plasma or whole blood and have used them as a vehicle for drug delivery. In this work, we bypassed the whole fibrinogen separation process and used blood or plasma directly to make a semisynthetic gel material. To this end, we did not rely on the thrombin for gelation but instead have added synthetic, biocompatible crosslinkers, which provide ability to tailor the sealant properties for a variety of biomedical applications. In this work, a series of quite inexpensive commercially available (4Arm-PEG-SG and PTE-050GS) and laboratory synthesized crosslinking agents (PluF68-NHS, PluF127-NHS, PEG-1000 NHS) were used to clot or crosslink plasma. Specifically, the gelation ability of these crosslinkers was tested on porcine plasma. The plasma gels had higher mechanical stability than the fibrin gels^35^, lower cytotoxicity and were capable of sustained drug release profiles. This approach enables use of autologous blood plasma to make surgical sealants and injectable drug delivery systems, eliminating the chance of antigenicity.

### Chemical characterization of the synthetic polymers

All the synthesized crosslinkers were characterized by FT-IR (Figs S1, S2) and ^1^H-NMR (Figs S3–S5).

PluF68-NHS: FT-IR (cm^−1^): 2882 (C-H aliphatic stretching), 1738 (N-hydroxysuccinimide (NHS) ester), 1336 (O-H bending), 1234 (C-N stretching), 1096 (C-O stretching); ^1^H-NMR: δ_H_ (400 MHz, CDCl_3_): ^1^H-NMR: δ_H_ (400 MHz, CDCl_3_): 1.141 (C*H*_3_, t), 1.970 (CH_2_-C*H*_2_-CH_2_, 4 H, dd), 2.432, (NHS-O-C(O)-C*H*_2_, 4 H, t), 2.723, (C*H*_2_ succinimide, 8 H, s), 2.846 (-CH2-C(O)-O, 4 H, q), 3.400 (CH_3_-C*H*, m), 3.557 (-O-CH(CH_3_)-C*H*_2_, s), 3.645 (C(O)-O-CH_2_-C*H*_2_, broad s), 4.247 (C(O)-O-C*H*_2_, 4 H, t).

PluF127-NHS: FT-IR (cm^−1^): 2878 (C-H aliphatic stretching), 1714 (NHS ester), 1340 (O-H bending), 1238 (C-N stretching), 1096 (C-O stretching); ^1^H-NMR: δ_H_ (400 MHz, CDCl_3_): 1.140 (C*H*_3_, t), 2.076 (CH_2_-C*H*_2_-CH_2_, 4 H, dd), 2.407, 2.427, 2.497, 2.511 (NHS-O-C(O)-C*H*_2_, 4 H, dd), 2.720, (C*H*_2_ succinimide, 8 H, s), 2.843 (-CH2-C(O)-O, 4 H, q), 3.405 (CH_3_-C*H*, m), 3.557 (-O-CH(CH_3_)-C*H*_2_, s), 3.645 (C(O)-O-CH_2_-C*H*_2_, broad s), 4.244 (C(O)-O-C*H*_2_, 4 H, t).

### Gelation study

**Figure 1:**
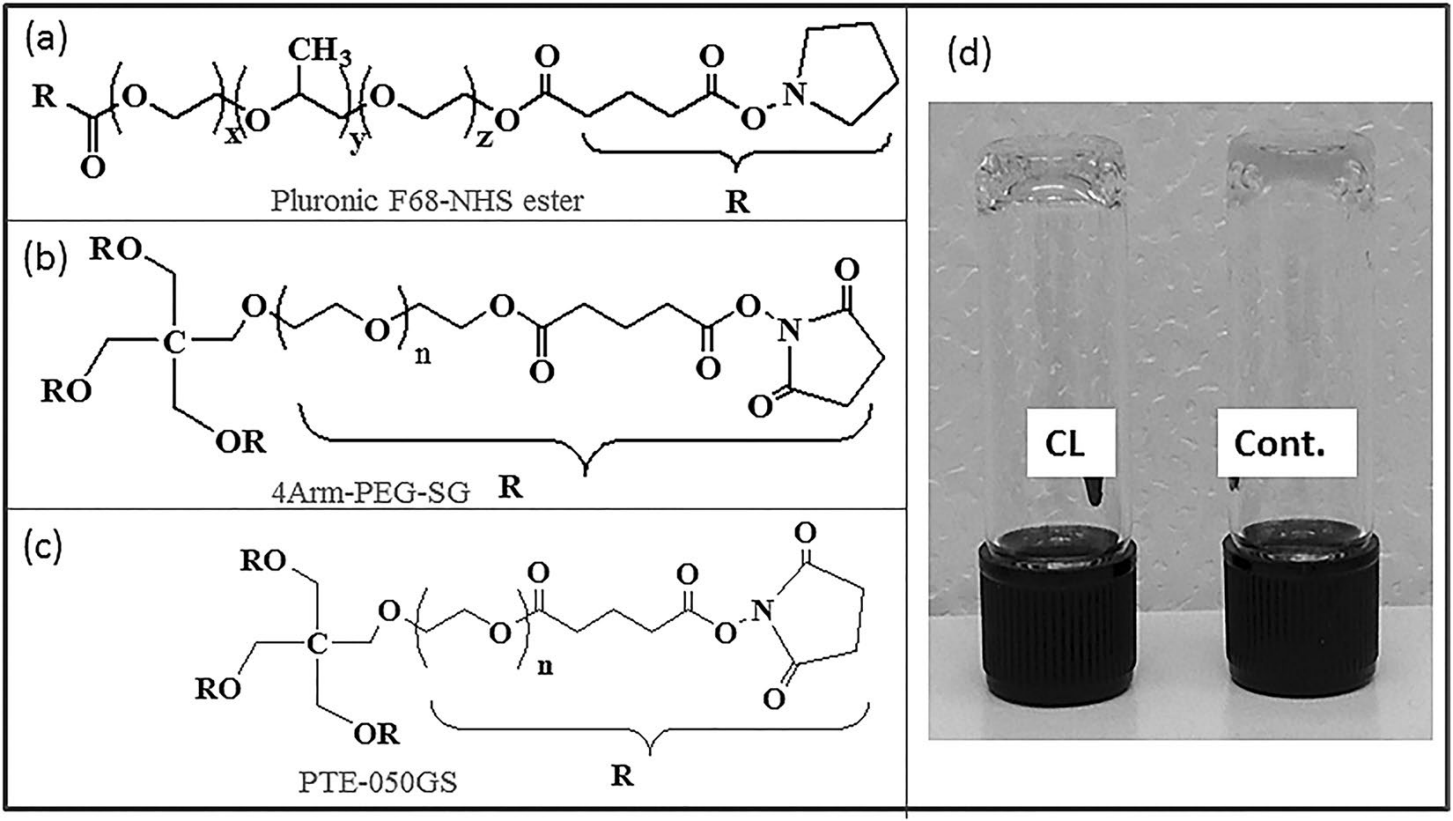
(**a**–**c**) Chemical structures of the synthetic crosslinkers which gelled porcine plasma. (**d**) Image of plasma gel formed by crosslinker (CL) and Ca(NO_3_)_2_(Cont.).

A gelation study of porcine plasma was carried out by using a number of biodegradable multifunctional (di, tri and tetra) NHS functionalized synthetic chemical crosslinkers. The crosslinkers were either synthesized in the lab as discussed in the ‘Method’ (PluF68-NHS, PluF127-NHS, PEG-1000 NHS) or obtained commercially (4Arm-PEG-SG and PTE-050GS). The crosslinker structures are presented in Fig. 1(a–c). Before using plasma, the gelation ability of the crosslinker solutions were at first tested in presence of 10% BSA solution in PBS. 10% BSA solution was chemically pure, single molecular weight protein which mimics the total protein content including albumin, globulin, fibrinogen etc. in the plasma^38^. All the crosslinker solutions were found to gel 10% BSA solution in less than 1 minute indicating their ability to crosslink with the protein. Commercially available blood/plasma, used for the study, contained organic salt like sodium citrate as anticoagulant which efficiently prevents blood clotting by chelation of calcium and other metal ions^39^, or natural anticoagulant like heparin which enhance the inhibitory activity of the plasma protein antithrombin^40^. Therefore, clotting or gelation ability of citrated plasma, was checked by adding 10% calcium nitrate (Ca(NO_3_)_2_) solution with the plasma in 1:20 ratio. Ca^+2^ ions have multiple roles in the coagulation of blood/plasma. Addition of Ca^+2^ ion result to the gelation of plasma by activating enzymes^41^, providing binding sites to the plasma proteins^42^ etc. The formed natural clot was also used as ‘control gel’ (Fig. 1d) during the measurement of properties as well as drug release experiments. To mimic the pH of the chronic wound region, pH of the plasma was elevated to ~8.0 by adding 1 μL of 20% solution of TEA per 10 μL of the plasma prior to mixing with the crosslinkers^43,44^. However, the crosslinkers were also able to gel plasma at its normal pH (pH 7.4). After confirming the gelation ability of the crosslinkers with the model solution and clotting ability of porcine plasma separately, gelation of porcine plasma was at first randomly tested with 20% solution of PluF68-NHS crosslinker solution in PBS in presence of TEA (pH~8) in different volume ratio. For this, 10 μL of the crosslinker solution was mixed with the porcine plasma in different volume ratios including 1:1, 1:2, 1:3, 1:4 and 1:5. It was observed that the crosslinker solution gelled plasma only in 1:1 and 1:2 ratios, forming a strong gel at 1:1 ratio and a weak gel at 1:2 ratio. Therefore, the gelation study of plasma with all the other crosslinker solutions in PBS (and DMSO) were carried out with the equivalent molar concentration of 20% PluF68-NHS, using one crosslinker at a time, where the strongest gel formation was observed when plasma and crosslinker solution were mixed in 1:1 volume ratio. A brief description of the concentrations of the crosslinkers used for the gelation is presented in Table S1. Being NHS functionalized, the crosslinkers crosslinked with the –NH_2_ groups in the proteins of plasma, resulting in translucent gels. Gelation test was carried out with the plasma at higher pH (pH~8) to mimic the pH of the chronic wound region. All the crosslinker solutions and gelation study was performed at room temperature except PluF127-NHS. This result is consistent with the previously reported literature where PluF127 solution transformed to gel at room temperature^45,46^. Plasma at pH 8 (in presence of TEA) was gelled by each of the above crosslinker solutions within 2 minutes to create translucent gels (Fig. 1d) which could be considered as ‘fairly quick’ for forming an *in situ* gel, acting as sealant or drug delivery platform However, the crosslinkers (PluF68-NHS and PTE-050GS) were able to gel plasma at its normal pH (7.4) in a much longer time ranging from 20 minute to 1 h. Gelation with PEG1000-NHS was inconsistent due to the low concentration and/or fast degradation of the crosslinker. The gels formed by each crosslinker visually appeared to be stronger than the control gel. All the gels were stable at room temperature as well as at body temperature (37 °C).

### Mechanical stability of the gels

**Figure 2:**
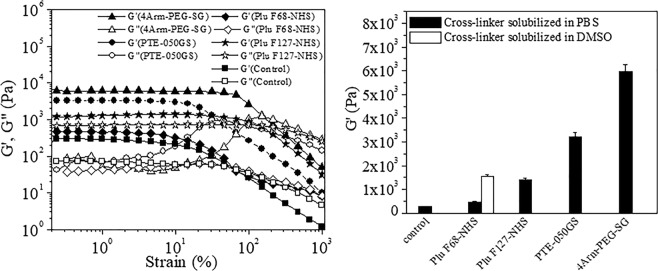
(**a**) Storage and loss modulus of the plasma gel by strain sweep measurement. (**b**) Representation of the storage modulus of all the plasma gels.

Viscoelastic property or mechanical stability of the gel was determined by oscillatory rheology measurement of the gels. The mechanical strength of the gel was measured using the storage modulus (G′) and loss modulus (G′′) and generally found dependent on the crosslinker concentration. Therefore, to compare the viscoelastic property of the gels, rheology measurements of the plasma gels were performed with the gels formed by the same molar equivalent concentration of the crosslinkers. Plasma gel formed by the addition of Ca(NO_3_)_2_ was used as control for the measurements. The plot in Fig. 2a indicated variation of G′ and G″ versus applied strain at a constant frequency of 1 Hz. The higher value of G′ than G″, up to the critical strain (the minimal strain required to partially break the network structure), indicated the viscoelastic nature of the gels. Above the critical strain value both G′ and G″ abruptly decrease. In the strain sweep experiment, all the gels showed their predominating elastic nature up to critical strain and then the viscous nature of the gel predominated indicating the destruction of the gel structure. As observed from the frequency sweep experiment, the G′ value was greater than G′′ at any given frequency, suggesting that the gels behave like solids (Fig. S6). Higher mechanical stability of the plasma gels formed by the crosslinkers compared to the control gel, was confirmed by the higher G′ values in both oscillatory strain and frequency sweep experiments. This is probably due to the availability of greater number of binding sites, which helps in forming greater entangled network by crosslinking NHS group of the crosslinkers and –NH_2_ group of the plasma protein. Gel strength was strongly dependent on the type and functionality (Fig. 2a,b) of the crosslinkers. The strength of the gels was found to be dependent on the functionality (bi or tetra) of the crosslinkers as indicated by the corresponding G′ values (Fig. 2b). The strength of the gels formed by tetra functional crosslinkers (4Arm-PEG-SG and PTE-050GS) was higher than bi-functional crosslinkers (PluF68-NHS and Plu F127-NHS) which was due to the higher number of binding sites available for crosslinking in tetra-functional crosslinkers. Also, the gels formed by the crosslinkers at pH 8 (in presence of TEA) had higher mechanical strength than the gels at pH 7.4 (in absence of TEA) as observed from their storage modulus value. The G′ value of the plasma gels at pH 7.4, formed by PluF68-NHS and PTE-050GS were found to be 55 Pa and 1550 Pa (Fig. S7) respectively whereas the respective values of the same gels at pH 8 were 460 Pa and 3230 Pa. Mechanical strength of plasma gel was also found to be dependent on the solvent which was used for the solubilization of the crosslinkers as indicated by the higher G′ value of the gels formed by DMSO solubilized PluF68-NHS than PBS solubilized PluF68-NHS, however the reason for this behavior is unknown. The higher storage modulus of these crosslinker formed plasma gels (1–6 kPa) than fibrin glue (0–0.15 kPa of fibrin conc:1–6 mg/mL)^34^ indicated its better mechanical stability for biomedical application. Frequency sweep data showed that the storage moduli of all the gels were almost independent of frequency at a range from 0.1–100 Hz indicating the existence of structured, solid-like material in the gel. The gel started to act as fluid-like material at the point it became frequency dependent.

### Cytotoxicity

Cytotoxicity of the crosslinkers was performed by live-dead assay and trypan blue exclusion study. For this study, mouse 3T3 cells were infused in the plasma gels formed by Ca(NO_3_)_2_ (control), PluF68-NHS (20%), 4Arm-PEG-SG (12%) and PTE-050GS (6%) crosslinker and kept in the incubator in presence of media. In a preliminary toxicity study, cells in the cell laden gels were cultured only for 1 h to check if the cells are surviving at all in the system, using both trypan blue and live-dead assay method. After that, cells in the cell laden gels were cultured for seven days and viability was measured on day 1, 4 and 7 using the live-dead assay method only.

In the preliminary study by trypan blue method, trypan blue was added after 1 h of culture to the cell laden gel and the cells were imaged under microscope (Fig. S8a–d). After imaging, all the cells were counted using Fiji ImageJ software. Cell viability in the control gel after 1 h was about 74%. 80–89% cell-viability (Fig. S8i) after 1 h of gel formation by the synthetic crosslinkers indicate non-toxicity of the crosslinkers. Preliminary live-dead assay was performed 1 h after the cell laden plasma gel preparation using the same crosslinkers and concentration as used in trypan blue method. The images of live (green) and dead (red) cells, infused in the plasma gels were shown in Figure A-L and Fig. S8e–h. 70% cell viability in the control gels and 75–88% (Fig. S8j) cell viability in synthetic crosslinkers gels again indicate non-toxicity of the crosslinkers.

**Figure 3:**
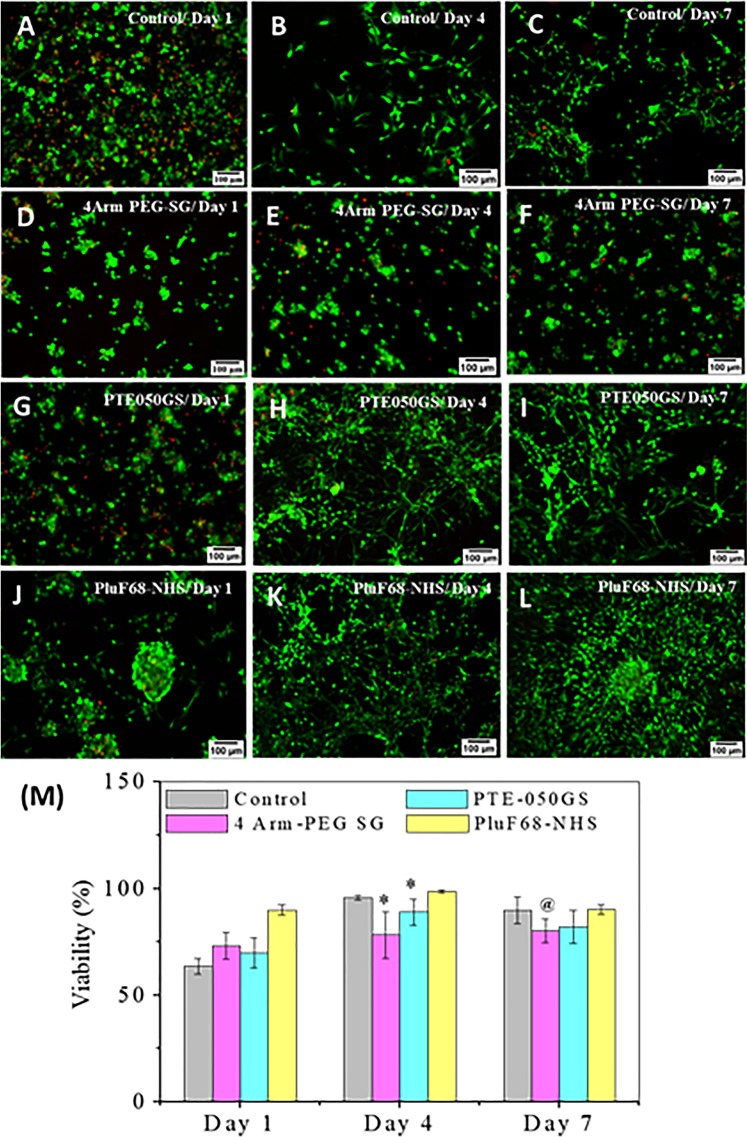
Cytotoxicity analysis of the crosslinkers using live-dead assay method of control (**A**–**C**), plasma gel by 4Arm-PEG-SG (**D**–**F**), plasma gel by PTE-050GS and (**G**–**I**) and plasma gel by PluF68-NHS (**J**–**L**) on day 1, 4 and 7. (**M**) Statistical analysis on the mean viability data of 3T3 cell in the plasma gel on day 1, 4 and 7.

After achieving promising data in the preliminary study, viability study was extended up to 7 days. The images of live (green) and dead (red) cells on day 1, 4 and 7 of culture were showed in Fig. 3A-3L and mean cell-viability in each type of gel were presented in Fig. 3M. Initially, after 1 day, cell viability in control gel was only 63% which recovered to a greater extent on day 4 and 7. On day 1, cell viability of the crosslinker gels was in between 73–90%. On day 4, cell viability in all the gel samples increased drastically showing 95% for the control gel and 78–98% for the crosslinker gels. Although the cell viability on day 7 was observed to be higher than on day 1 but it is slightly less than that on day 4. This might be due to the over-population of cells caused by proliferation for a longer time in the samples. Cytotoxicity analysis of the crosslinkers for an extended time indicated their less toxic nature, which was ideal for their biomedical applications. Statistical analysis of all the viability data of control and all crosslinker gels on day 1, 4 and 7 was performed individually by one-way ANOVA. On day 1 and day 4, there were statistically significant differences of the mean viability between all the four groups including control, 4Arm-PEG-SG, PTE-050GS, and PluF68-NHS as indicated by the p value (day 1: p = 0.00238 < 0.05; day 4: p = 0.00846 < 0.05) but on day 7 the difference of mean viability between the groups was not significant. After that, two tail Student’s t test was performed with control and each crosslinker gel and the results were summarized in Fig. 3M. On day 1, there was no significant difference between the mean viability of control and 4Arm-PEG-SG (p = 0.17425 > 0.05); control and PTE-050GS (p = 0.28637 > 0.05) and control and PluF68-NHS (p = 0.09636 > 0.05). On day 4, although there was no significant difference between the mean viability of control and PluF68-NHS (p = 0.27209 > 0.05), but the mean viability of control and 4Arm-PEG-SG (p = 0.00374 < 0.05) and control and PTE-050GS (p = 0.03599 > 0.05) were significantly different. On the other hand, on day 7, there was no significant difference between the mean viability of control and PTE-050GS (p = 0.10152 > 0.05) and control and PluF68-NHS (p = 0.91458 > 0.05), but the mean viability of control and 4 Arm-PEG SG (p = 0.03856 < 0.05) were significantly different.

### Biodegradation

For this study, two types of enzyme, collagenase and pepsin were selected. Collagenases are enzymes that break the peptide bonds in collagen. Biodegradation was studied with a wide range of enzyme concentration including 0.5%, 1% and 2% with the plasma gels formed by PluF68-NHS, PluF127-NHS, 4Arm-PEG-SG and PTE-050GS, along with the control gel. All the gels were observed to be digested in the enzyme solutions indicating their biodegradability in the presence of enzyme. The degradation time of the gel could be altered with the concentration of the enzymes. Although, degradability was observed in the highest concentration of enzyme (2%) within 2–7 days, it took several weeks to be digested in the lower concentrations of the enzyme. The digestion time of the gels are presented in Table 1. It was observed that control gels were digested in a much lower time than the crosslinker gels. On the other hand, gels formed by tetra-functional crosslinkers took longer time to digest than the gels formed by bi-functional crosslinker, suggesting the formation of stronger gels with the tetra-functional crosslinker. Plasma gels formed by 4Arm-PEG-SG were not observed to digest in 0.5% of the enzyme solutions after 15 days of the start of experiment. The time of the enzyme degradation of the plasma gel correlates their mechanical stability values. Although all the gels were biodegradable, crosslinker formed plasma gels took much longer time to digest in both the enzymes compared to the control gel, which might be due to their higher mechanical stability as obtained from the rheology.

**Table 1:** Enzymatic degradation time for the plasma gels.

	Collagenase			Pepsin		
	0.5%	1%	2%	0.5%	1%	2%
Control	96 h	51 h	20 h	78 h	25 h	5 h
PluF68-NHS	140 h	68 h	21 h	136 h	35 h	15 h
PluF127-NHS	290 h	120 h	29 h	240 h	76 h	18 h
PTE-050GS	335 h	145 h	96 h	160 h	120 h	22 h
4Arm-PEG-SG	—	191 h	168 h	—	150 h	22 h

### Sterility study

Sterility of the plasma was tested to investigate if the plasma is free from organisms. Plasma was mixed aseptically with TSB in 1:100 ratio and incubated at 37 °C with lid left slightly loose to facilitate the culture of aerobic bacteria. TSB-only negative controls (no plasma) were kept in the same way to confirm sterility and lack of self-contamination. Both control and plasma/TSB mixture were observed every day for 7 days to check if there is any growth in the medium. No growth or changing turbidity was visually observed in any of the medium which indicated that the plasma was sterile.

### Drug release experiment

**Figure 4:**
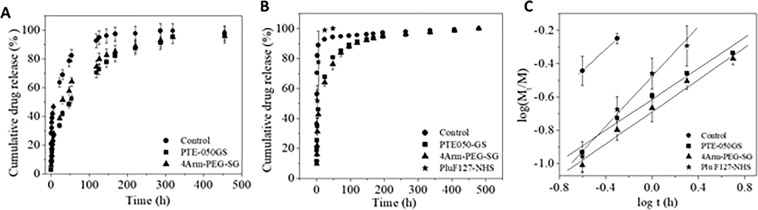
Cumulative release profile of (**A**) blue dextran and (**B**) rifampin in PBS from control plasma gel and crosslinker formed plasma gel. (**C**) Rifampin release profile up to 60%, indicating its linear relation with release time.

The higher mechanical stability than the fibrin gels^34^, lower cytotoxicity and biodegradability made it a suitable drug carrier in the wound. It was hypothesized that upon application of drug infused crosslinker solution over the wound, the plasma/blood will be gelled immediately along with the drug and the trapped drug will be released slowly (pH 7.4) by diffusion from the gel. Therefore to mimic the drug release *in vitro*, at first, 5 wt% blue dextran infused plasma gel (400 μL) was prepared using Ca(NO_3_)_2_ (control gel), 4Arm-PEG-SG and PTE-050GS crosslinkers in a capped vial and PBS (pH 7.4) was used as the release medium. Drug loaded PBS was then collected completely and its absorbance were measured at various time intervals. The release profile of blue dextran (Fig. 4A) indicated that in first 30 minutes a burst of 20% drug was released from the control gel where 3–7% drug was released from the crosslinker induced gel. At the end of the drug release where almost 90% drug was released, it was observed that the crosslinker induced gel took about 290 h while the control gel took only 190 h. Then drug release study was carried out with rifampin, an antibiotic drug, from plasma gel prepared with Ca(NO_3_)_2_ (control gel), PluF127-NHS, 4Arm-PEG-SG and PTE-050GS. The experiment procedure was same as done for blue dextran. Rifampin release was faster than blue dextran which might be due to its lower molecular weight which expeditated its delivery from the gel to PBS. Initially, within first 30 minutes, almost 60% rifampin had released from control gel and the release got over within 1 day (Fig. 4B). On the other hand, crosslinker gels took comparatively much longer time to release rifampin. In these cases, only 15–20% drug was released in first 30 min. Although it took 24 h to release 60% rifampin from the plasma gel made with 4Arm-PEG-SG and PTE-050GS, the time was substantially less in case of plasma gel made with PluF127-NHS, only 2 h. Therefore, it can be concluded that the drug release from the synthetic crosslinker induced gel was slower and more sustained than the control gel. The rifampin release profile, up to 60%, was analyzed using Eq. (1) (Fig. 4C). Table 2 presents the n and k-values from Eq. (1) for the rifampin release from all the plasma gels. n value of 0.5 suggests normal Fickian diffusion; n of 1.0 indicates time-independent zero-order release and n value in between 0.5 and 1.0 indicate that there is some anomalous or Non-Fickian, diffusion taking place in the system^47^. The n values in Table 2 indicated Fickian diffusion of drug from the plasma gel prepared with 4Arm-PEG-SG and PTE-050GS but from control and PluF127-NHS gel, the diffusion was anomalous. The n values also suggested that none of the release followed zero-order release kinetics.

**Table 2:** n and k values of the 60% rifampin release from plasma gels.

	n	logk	R^2^
Control	0.64	−0.0564	1
PTE-050GS	0.46	−0.6143	0.98
4Arm-PEG-SG	0.48	−0.68919	0.99
PluF127-NHS	0.75	−0.47606	0.99

## Conclusions

In summary, we have developed an alternative to fibrin glue sealant materials wherein blood plasma proteins can be crosslinked using biocompatible water soluble crosslinkers to form crosslinked gels. The gelation reaction occurs quickly in few seconds to minutes. All the formed plasma gels are nontoxic to mammalian cells and its mechanical properties are tunable by the type of crosslinker used. All the gels were observed to be biodegradable in presence of enzyme. Nontoxicity, biodegradability and higher mechanical stability of the plasma gels depict its promising use as drug release carrier. Drug release studies indicated its controlled release profile which can be tuned for a given biomedical application. Therefore, on application of the crosslinker solution in the wound is expected to clot blood immediately, resulting, lesser blood loss. Unlike most of the surgical sealants, which are mostly two separate products that must be mixed and then delivered in a specially designed system, it is one component simple system which can be applied easily on the wound. On the other hand, on application of the drug infused crosslinker solutions will produce drug loaded blood clot where the sustained release of drugs can result quick wound healing.

## Methods

### Synthesis of di-functional crosslinker PluF127-NHS ester and PluF68-NHS ester

**Figure 5:**
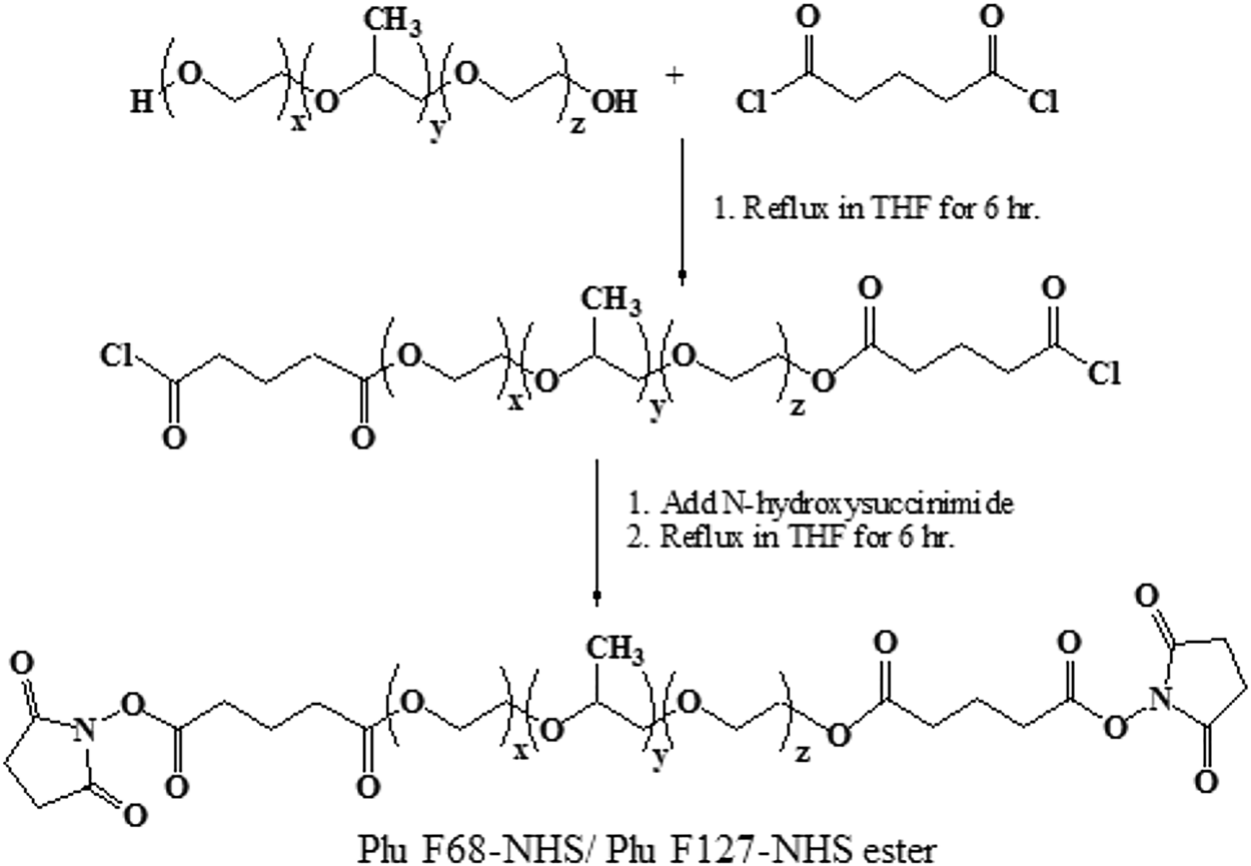
Reaction synthetic scheme of PluF68 NHS/PluF127-NHS ester.

10 g of Pluronic F127 (PluF127) (Spectrum, USA) or Pluronic ® F68 (PluF68) (Sigma-Aldrich, USA) was dried under vacuum for 1 h at 110 °C. In a 250 mL two necked flask equipped with a reflux condenser and nitrogen inlet, glutaryl chloride (Sigma-Aldrich, USA) (5eq, 0.57 mL) was dissolved in 30 mL dry THF. While rapidly stirring the glutaryl chloride solution using a magnetic stir bar, PluF127 or PluF68 (10 g), dissolved in 30 mL dry THF, was added dropwise, followed by 0.91 mL (5 eq) triethylamine (Sigma-Aldrich, USA). The reaction mixture was then refluxed under positive nitrogen atmosphere for 3 h, cooled to room temperature and then NHS (5 eq, 1.04 g), dissolved in 20 mL dry THF, was added to the reaction mixture. The mixture was refluxed for another 3 h. The reaction mixture was then cooled to room temperature and filtered to remove triethylamine hydrochloride. The filtrate was added to 1 L ice-cold hexane to precipitate PluF127-NHS ester or PluF68-NHS ester as a white solid. The product was dried under vacuum (yield: ~10 g). The reaction scheme is presented in Fig. 5.

### Synthesis of tri-functional crosslinker PEG1000-NHS ester

10 g of Glycerol-PEG 1000 triol was dried under vacuum for 1 h at 90 °C. In a 250 mL two-necked flask equipped with a reflux condenser and nitrogen inlet, glutaryl chloride (5eq, 3.8 mL) was dissolved in 30 mL dry tetrahydrofuran (THF). While rapidly stirring the glutaryl chloride solution using a magnetic stir bar, PEG 1000 triol (5 g), dissolved in 30 mL dry THF, was added dropwise, followed by 5.2 mL triethylamine. The reaction mixture was refluxed under positive nitrogen atmosphere for 3 h, cooled to room temperature and then 1.73 g N-hydroxysuccinimide, dissolved in 20 mL dry DCM, was added to the reaction mixture. The mixture was refluxed for another 3 h. The reaction mixture was then cooled to room temperature and filtered to remove triethylamine hydrochloride. The filtrate was concentrated and added dropwise into one-liter ice-cold diethyl ether to precipitate brown semi solid PEG1000-NHS ester. The semi solid product phase was separated from the diethyl ether and dried under vacuum. The reaction scheme is presented in Fig. S9.

### General instrumentation

The ^1^H-NMR spectra were recorded on a Varian 400 MHz instrument in CDCl_3_ solvent with TMS as a reference standard. The FT-IR spectra were measured with a ThermoNicolet Nexus 470 FT-IR (Model 470) spectrometer. A drug release experiment was monitored by using a FLUOstar Omega BMG LABTECH UV-Vis spectrophotometer. Cell viability was determined with the trypan blue method using a Nikon (Model:TMS-F, Tokyo) microscope and with the live-dead method using a Leica DMI 6000B fluorescent microscope and Zeiss (Observer Z1, Germany) inverted microscope equipped with ApoTome.2 (Zeiss, Germany). All measurements were performed at room temperature (25 °C) unless otherwise mentioned.

### Gelation test

Gelation of porcine plasma (Animal Technologies, Texas, USA) was studied with PluF68-NHS ester crosslinker solution in PBS and DMSO, and with PluF127-NHS ester, 4Arm-PEG-SG (Laysan Bio, Inc. Alabama, USA) and PTE-050GS (NOF Corporation, Tokyo, Japan) crosslinker solutions in PBS at different concentrations and volume ratios in presence of 1 μL of 20% solution of triethanol amine (TEA) per 10 μL of the plasma. For this study, crosslinker solutions of PluF68-NHS ester, 4Arm-PEG-SG and PTE-050GS were prepared in PBS (or DMSO) and mixed with plasma at different volume ratios of crosslinker to plasma while maintaining the mixtures at room temperature. Solid PluF127 NHS ester was dissolved in PBS at 0 °C, in an ice bath and maintained at the same 0 °C temperature throughout the gelation study with plasma. In all cases, the respective crosslinked gels were formed in less than 2 minutes. Additionally, a standard model solution of 10% bovine serum albumin (BSA) was prepared in PBS to verify gelation capabilities of the the crosslinker solutions under study in a 1:1 volume ratio.

### Mechanical stability

Mechanical stability of each crosslinked gel was measured by rheology at room temperature using a parallal plate rheometer with PP25 as the plate geometry and maintaining a 0.2 mm plate gap. For the measurement, 0.15 mL crosslinker solution was prepared, mixed with equal volume of plasma (pH 8, maintained by TEA), and then immediately cast on the rheometer plate. The gel was formed in <2 minutes. For each measurement, the viscoelastic region was found from a strain sweep (strain range 0.01–1000%) measurement at a fixed frequency of 1 Hz. After that, frequency sweep measurement was performed within a wide range of frequency of 0.01–100 Hz at a fixed stain of 1% chose from the linear viscoelastic region determined with the stain sweep measurement.

### Enzymatic degradation

For the enzymatic degradation/biodegradation studies, 100 μL of plasma gels for each crosslinkers were prepared including controls. All gels were incubated in 1 mL of collagenase (Sigma-Aldrich, USA) solution in PBS and pepsin (Sigma-Aldrich, USA) solution in 0.1 N HCl with different concentrations at 37 °C. The degradation of the gels was evaluated every hour.

### Cytotoxicity

Toxicity of the crosslinked gels was determined by both trypan blue (HyClone Laboratories, Utah, USA) method and live-dead assay method using a Live/Dead assay kit (Life technologies, USA), following manufacturer protocol. Mouse 3T3 cells were used with cell density of 10 million/mL for both methods. Cells were cultured in DMEM media with 10% FBS for 5 days. Each crosslinker solution was prepared in PBS and sterile filtered. Plasma (pH 8, maintained by TEA) was sterile filtered separately. To make the cell laden gel samples, at first cells were trypsinized, counted and mixed with sterile plasma. Individual samples were prepared by mixing 10 μL cell infused plasma and 10 μL crosslinker solution on a 12 mm glass coverslip. Within approximately 1 minute, the mixture turned to a gel, then the coverslips were placed in a 24 well plate with culture media and kept in the incubator. For both trypan blue and live-dead assay method, three individual samples of mouse 3T3 cell (density 10 million/mL) laden plasma gels for each crosslinker were prepared. For the trypan blue method, trypan blue was added to the gel samples after 1 h of culture, and the cells were counted under microscope (Nikon TMS-F). For live-dead method, the cell laden gel samples were stained after 1 h for the preliminary study and on day 1, day 4 and day 7 for the extended viability study. Then Zstack fluorescent images of the stained cells were acquired using an inverted microscope. The viability percentage was defined as number of viable cells (green) divided by total number of cells (total of green and red).

### Sterility study

Plasma was filtered through a 0.2 μm membrane filter. 3% tryptic soy broth (TSB) was prepared by dissolving TSB powder in DI water and sterilized. At first, 2 mL TSB was taken in a sterile screw capped glass tube and then 20 μL plasma was mixed aseptically in it. The glass tubes were then incubated at 37 °C with lid left slightly loose to let the air pass. 2 mL of 3% TSB, without any plasma was also kept in the same way which served as negative control. All the samples were observed every day for 7 days to check if there is any growth in the medium.

### Drug release experiments

Drug release experiments were carried out at first with blue dextran (M.W. 20,000) (Sigma, USA) and then with rifampin (Gold Biotechnology, Missouri, USA) (M.W. 823) loaded in the plasma gels, formed by crosslinkers as well as control plasma gels (formed only in the presence of calcium ion, without any crosslinker). 5 wt% blue dextran loaded 4Arm-PEG-SG (12 wt%) and PTE-050GS (6 wt%) crosslinker solutions and 1 wt% rifampin loaded PluF127-NHS (28.6 wt%), 4Arm-PEG-SG (12 wt%) and PTE-050GS (6 wt%) crosslinker solutions were prepared in PBS. 200 μL of the drug infused crosslinker solutions were mixed with 200 μL porcine plasma (pH~8) in a 4 mL screw-capped vial where the mixture gelled in 1 minute. Drug loaded plasma gels were kept at 37 °C and then 2 mL PBS was gently added on the top without disturbing the gel. The supernatant above the gel was removed and replaced at various time intervals. Amount of drug release was measured by monitoring its intensity with UV-Vis spectrometer. The rifampin release profile was analyzed to determine dependence of the release on drug loading and time using equation.

1 $${{\rm{M}}}_{{\rm{t}}}/{\rm{M}}={{\rm{kt}}}^{{\rm{n}}}$$

where M_t_/M is the fraction of total rifampin released at time t, t is the release time, k is the rate constant dependent on the system geometry and diffusion coefficients^47^, n is the exponent of the power function which denotes the order of the reaction. The n-values from Eq. (1) were calculated by linear regression of Eq. (2) using least squares estimates.

2 $$\mathrm{log}\,{{\rm{M}}}_{{\rm{t}}}/{\rm{M}}={\rm{nlog}}\,{\rm{t}}+\,\mathrm{log}\,{\rm{k}}$$

### Statistical analysis

The results for viability and drug release were reported as mean ± standard deviation (SD). The viability data was analyzed using Student’s t-test and ANOVA statistical methods. For the overall statistical analysis between all the groups, one-way ANOVA was used, with a p-value < 0.05 considered to be significant. To determine a statistically significance difference between the groups, we used two tail Student’s t comparison test, with a p-value < 0.05 considered to be significant. All the statistical analyses were performed in Microsoft Excel 2016.

## Supplementary information

Supporting Info

## Data Availability

All data including images and graphs, generated during this study are included in this article (and its Supplementary Information Files. Statistical analysis data generated during study are available from the corresponding author on request.

## References

[1] Peppas NA, Langer R (1994). New challenges in biomaterials. Science.

[2] Madan M, Bajaj A, Lewis S, Udupa N, Baig J (2009). *In situ* forming polymeric drug delivery systems. Indian J. Pharm. Sci..

[3] Liow SS (2016). Thermogels: *In Situ* Gelling Biomaterial. ACS Biomater. Sci. Eng..

[4] Deepthi S, Jayakumar R (2018). Alginate nanobeads interspersed fibrin network as *in situ* forming hydrogel for soft tissue engineering. Bioact. Mater..

[5] Li Y, Meng H, Liu Y, Lee BP (2015). Fibrin Gel as an Injectable Biodegradable Scaffold and Cell Carrier for Tissue Engineering. *Sci*. World J..

[6] Dhawan S, Kapil R, Kapoor DN (2011). Development and Evaluation of *In Situ* Gel-forming System for Sustained Delivery of Insulin. J. Biomater. Appl..

[7] Kotreka UK, Davis VL, Adeyeye MC (2017). Development of topical ophthalmic *In Situ* gel-forming estradiol delivery system intended for the prevention of age-related cataracts. PLoS One.

[8] Liu L, Gao Q, Lu X, Zhou H (2016). *In situ* forming hydrogels based on chitosan for drug delivery and tissue regeneration. Asian J. Pharm. Sci..

[9] Kouchak M (2014). *In situ* gelling systems for drug delivery. Jundishapur J. Nat. Pharm. Prod..

[10] Ko DY, Shinde UP, Yeon B, Jeong B (2013). Recent progress of *in situ* formed gels for biomedical applications. Prog. Polym. Sci..

[11] Xie X (2013). A novel hemostatic sealant composed of gelatin, transglutaminase and thrombin effectively controls liver trauma-induced bleeding in dogs. Acta Pharmacol. Sin..

[12] Behrens AM (2015). Biodegradable-Polymer-Blend-Based Surgical Sealant with Body-Temperature-Mediated Adhesion. Adv. Mater..

[13] Liu Y (2009). Biomimetic sealant based on gelatin and microbial transglutaminase: An initial *in vivo* investigation. J. Biomed. Mater. Res. Part B Appl. Biomater..

[14] Brubaker CE, Messersmith PB (2011). Enzymatically Degradable Mussel-Inspired Adhesive Hydrogel. Biomacromolecules.

[15] Songkroh T (2015). Erratum to: *In situ* forming chitosan-based hydrogel as a lung sealant for biological lung volume reduction. Sci. Bull..

[16] Barrett DG, Bushnell GG, Messersmith PB (2013). Mechanically Robust, Negative-Swelling, Mussel-Inspired Tissue Adhesives. Adv. Healthc. Mater..

[17] Pinkas O, Zilberman M (2017). Novel gelatin–alginate surgical sealants loaded with hemostatic agents. Int. J. Polym. Mater. Polym. Biomater..

[18] Burnouf T (2013). Blood-derived biomaterials and platelet growth factors in regenerative medicine. Blood Rev..

[19] Murphy KC, Leach JK (2012). A reproducible, high throughput method for fabricating fibrin gels. BMC Res. Notes.

[20] Matthias N (2018). Volumetric muscle loss injury repair using *in situ* fibrin gel cast seeded with muscle-derived stem cells (MDSCs). Stem Cell Res..

[21] Janmey PA, Winer JP, Weisel JW (2009). Fibrin gels and their clinical and bioengineering applications. J. R. Soc. Interface.

[22] Huang Y-C, Dennis RG, Larkin L, Baar K (2005). Rapid formation of functional muscle *in vitro* using fibrin gels. J. Appl. Physiol..

[23] Liu J (2012). Soft fibrin gels promote selection and growth of tumorigenic cells. Nat. Mater..

[24] Chernysh IN, Nagaswami C, Purohit PK, Weisel JW (2012). Fibrin clots are equilibrium polymers that can be remodeled without proteolytic digestion. Sci. Rep..

[25] Burnouf T, Su C-Y, Radosevich M, Goubran H, El-Ekiaby M (2009). Blood-derived biomaterials: fibrin sealant, platelet gel and platelet fibrin glue. ISBT Sci. Ser..

[26] Mintz PD (2001). Fibrin sealant: clinical use and the development of the University of Virginia Tissue Adhesive Center. Ann. Clin. Lab. Sci..

[27] Spotnitz WD, Prabhu R (2005). Fibrin sealant tissue adhesive–review and update. J. Long. Term. Eff. Med. Implants.

[28] Parazzi V (2015). Extensive Characterization of Platelet Gel Releasate from Cord Blood in Regenerative Medicine. Cell Transplant..

[29] Piccin A (2017). Platelet gel: a new therapeutic tool with great potential. Blood Transfus..

[30] Cheng K (2012). Intramyocardial Injection of Platelet Gel Promotes Endogenous Repair and Augments Cardiac Function in Rats With Myocardial Infarction. J. Am. Coll. Cardiol..

[31] Cheng K (2012). Transplantation of platelet gel spiked with cardiosphere-derived cells boosts structural and functional benefits relative to gel transplantation alone in rats with myocardial infarction. Biomaterials.

[32] Valbonesi M (2004). Fibrin glue and cryo-platelet gel for surgical application in Italy. Transfus. Apher. Sci..

[33] Kazemzadeh-Narbat M, Annabi N, Khademhosseini A (2015). Surgical sealants and high strength adhesives. Mater. Today.

[34] Wheat JC, Wolf JS (2009). Advances in Bioadhesives, Tissue Sealants, and Hemostatic Agents. Urol. Clin. North Am..

[35] Ryan EA, Mockros LF, Weisel JW, Lorand L (1999). Structural Origins of Fibrin Clot Rheology. Biophys. J..

[36] Bhagat V, Becker ML (2017). Degradable Adhesives for Surgery and Tissue Engineering. Biomacromolecules.

[37] Barreda L (2019). Human plasma gels: Their preparation and rheological characterization for cell culture applications in tissue engineering. J. Mech. Behav. Biomed. Mater..

[38] Busher, J. T. *Serum Albumin and Globulin*. *Clinical Methods: The History*, *Physical*, *and Laboratory Examinations*.*3rd edition*. (1990). 21250045

[39] Mikaelsson M. E. (1991). The Role of Calcium in Coagulation and Anticoagulation. Coagulation and Blood Transfusion.

[40] Gray Elaine, Hogwood John, Mulloy Barbara (2011). The Anticoagulant and Antithrombotic Mechanisms of Heparin. Heparin - A Century of Progress.

[41] Riddel JP, Aouizerat BE, Miaskowski C, Lillicrap DP (2007). Theories of Blood Coagulation. J. Pediatr. Oncol. Nurs..

[42] Weisel JW, Litvinov RI (2013). Mechanisms of fibrin polymerization and clinical implications. Blood.

[43] Gethin G (2007). The significance of surface pH in chronic wounds. Wounds UK.

[44] Sirkka, T., Skiba, J. B. & Apell, S. P. Wound pH depends on actual wound size. 1–13 (2016).

[45] Callan, M., Jang, E., Kelly, J. & Nguyen, K. Characterization of Pluronic F127 for the controlled drug release vancomycin in the spinal column. *J*. *Undergrad*. *Chem*. *Eng*. *Res*. Ed. 9–19 (2017).

[46] Ron & Bromberg (1998). Temperature-responsive gels and thermogelling polymer matrices for protein and peptide delivery. Adv. Drug Deliv. Rev..

[47] Vernon BL, Fusaro F, Borden B, Roy KH (2004). Partition-controlled progesterone release from waterborne, *in situ*-gelling materials. Int. J. Pharm..

