# Supplementary material for: Plasma-based fast-gelling biohybrid gels for biomedical applications
Source: Sci Rep. 2019 Jul 26;9:10881. doi: 10.1038/s41598-019-47366-3 (PMC6659638; doi:10.1038/s41598-019-47366-3)
Supplement: Supplementary file 1 — Supporting Info [file 41598_2019_47366_MOESM1_ESM.docx]

**Plasma-based fast-gelling biohybrid gels for biomedical applications**

Amrita Pal, Kunal Tripathi, Chandrashekhar Pathak and Brent L Vernon*

Arizona State University, Tempe, AZ 85287, USA


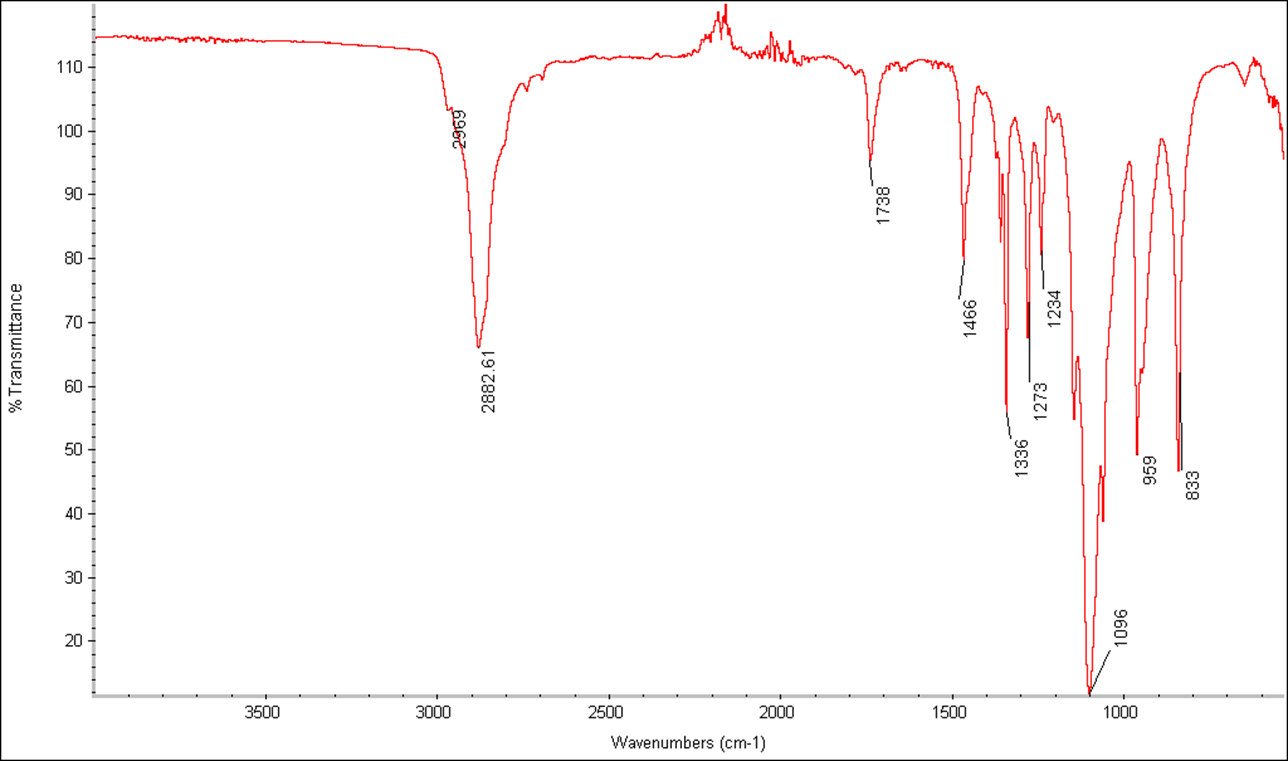


**Figure S1** FTIR of Plu F68-NHS.

**
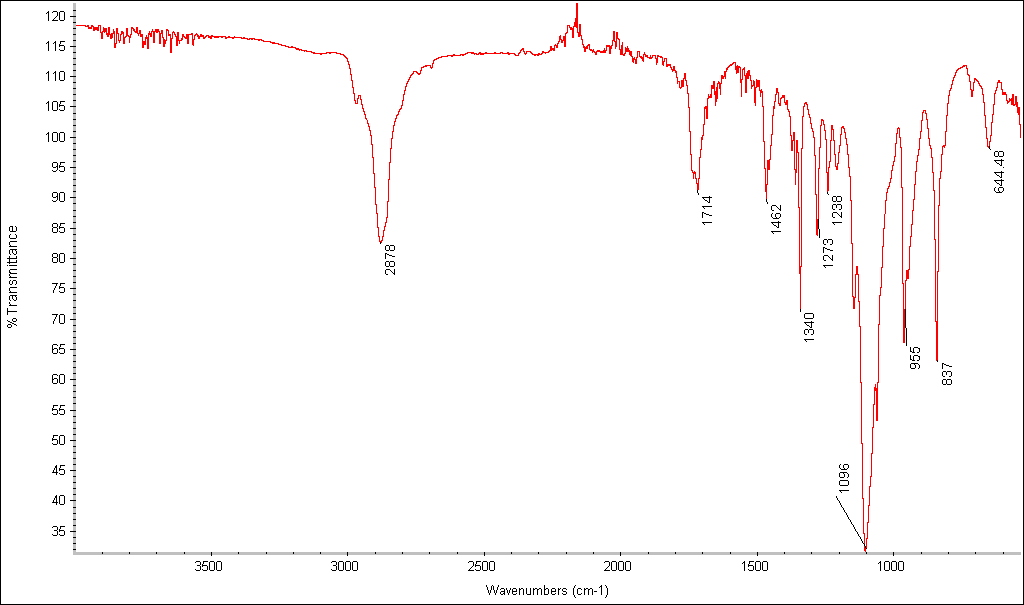
**

**Figure S2** FTIR of Plu F127-NHS.


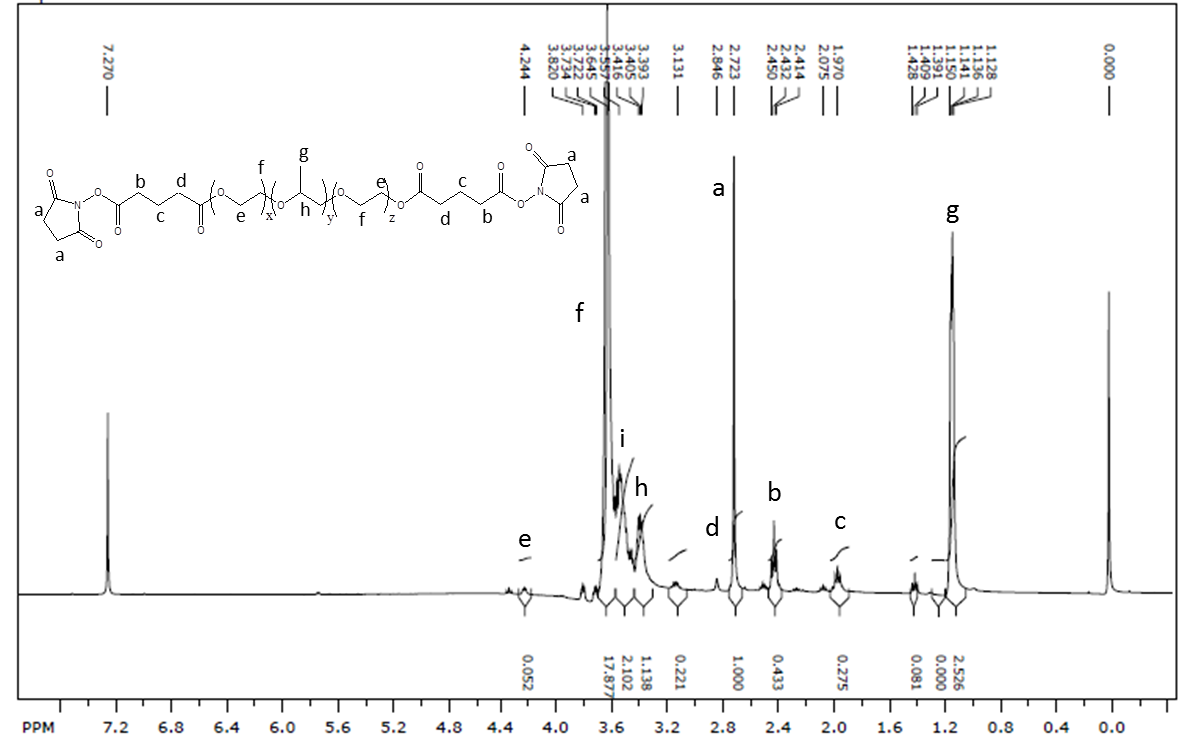


**Figure S3** NMR of Plu F68-NHS in CDCl_3_.


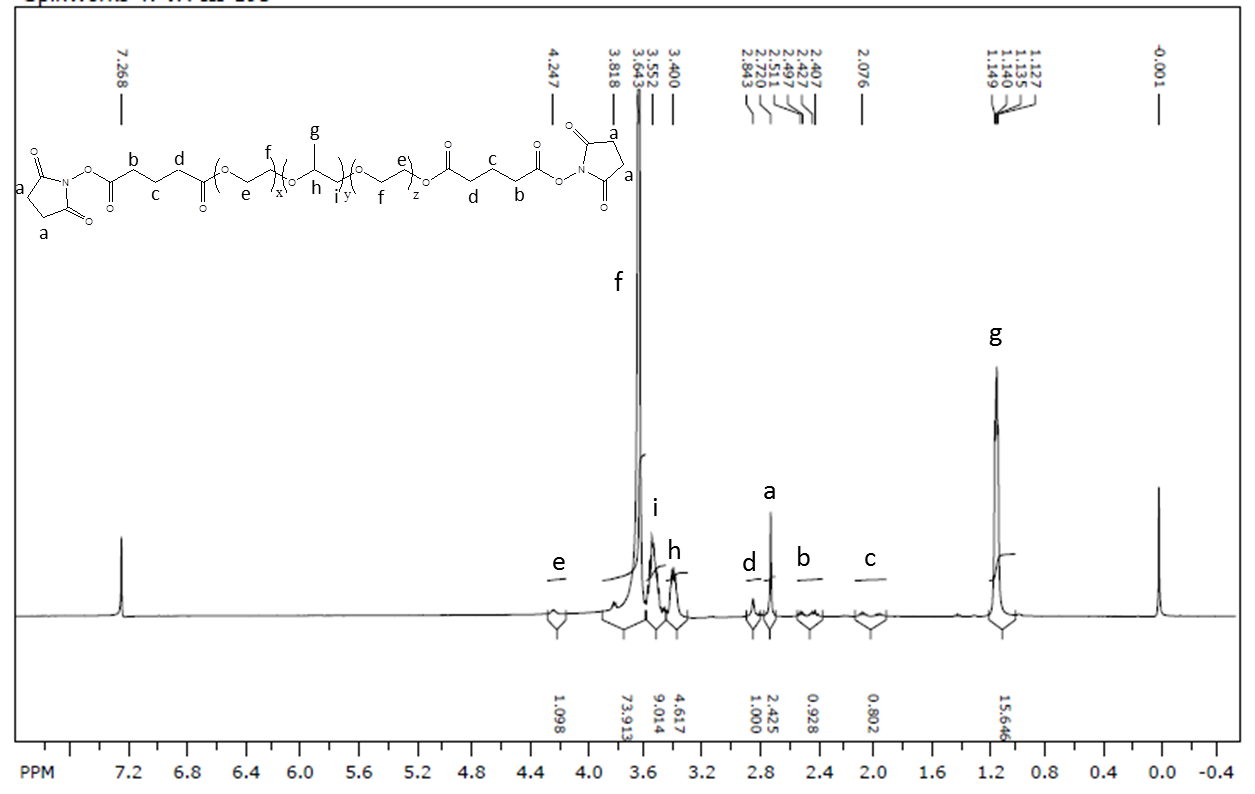


**Figure S4** NMR of Plu F127-NHS in CDCl_3_.


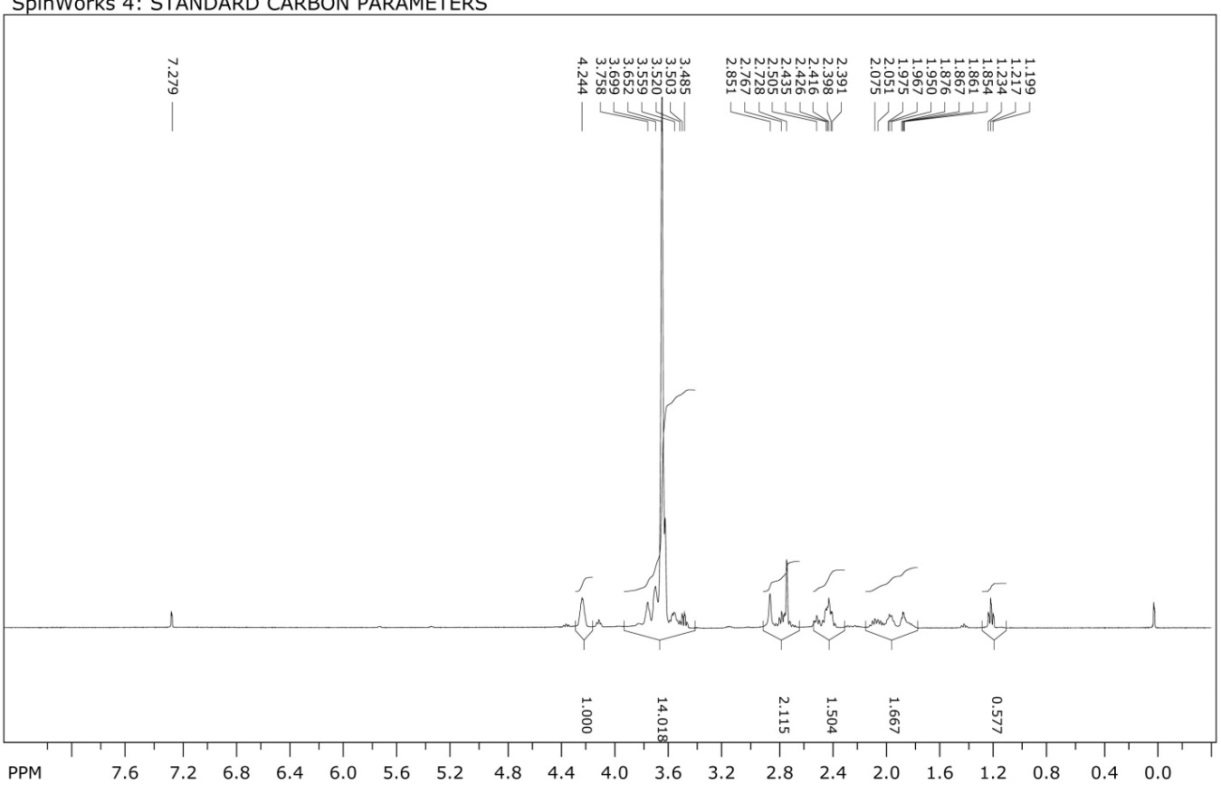


a

c, d

b

b

a

a

c

c

dc

dc

dc

edc

fdc

gdc

hdc

hdc

ha

h

f

e

g

f

e

g

a


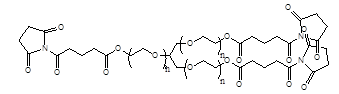


e, f

g

h

**Figure S5** NMR of PEG-1000 NHS in CDCl_3_.

Table S1. Summary of gelation study of plasma with synthetic crosslinkers with the volume ratio 1:1 (Plasma: Crosslinker).

| **Crosslinker** | **Concentration** | **Solvent** | **Status** |
| --- | --- | --- | --- |
| PTE-050GS | 6 wt% | PBS | Gel |
| 4Arm-PEG-SG | 12 wt% | PBS | Gel |
| PluF68-NHS | 20 wt% | PBS and DMSO | Gel |
| PluF127-NHS | 28.6 wt% | PBS | Gel |
| PEG-1000 NHS | 2.4 wt% | PBS | Gel (non- reproducible) |


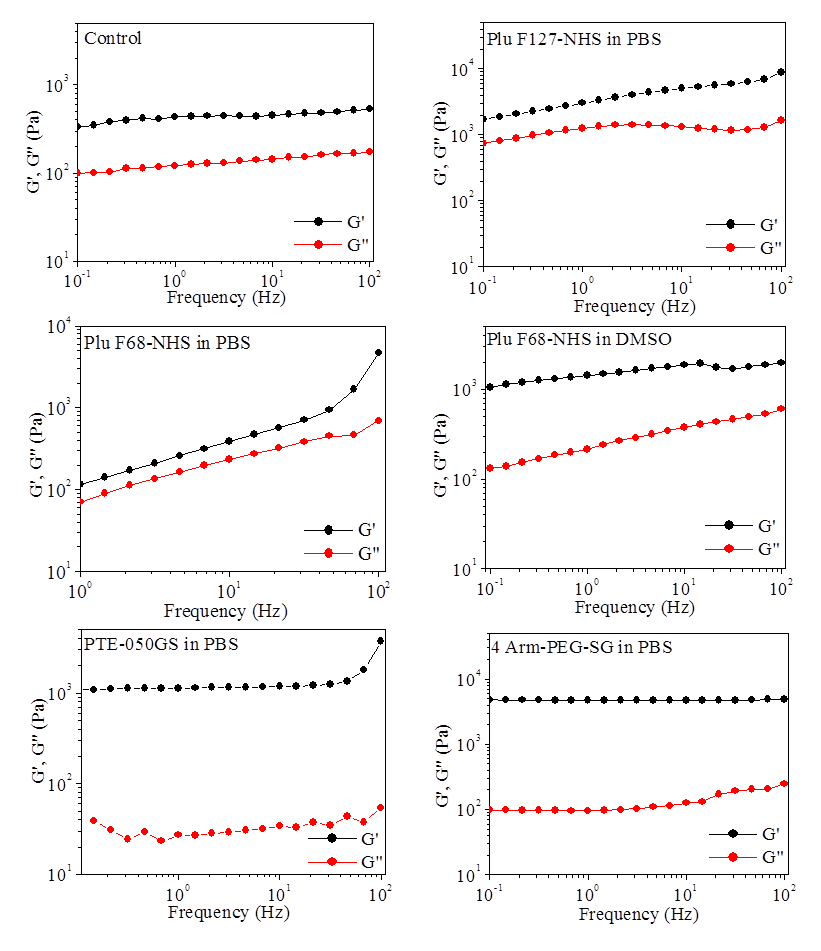


**Figure S6** Variation of storage and loss modulus of plasma gels with frequency at a constant strain of 1%.


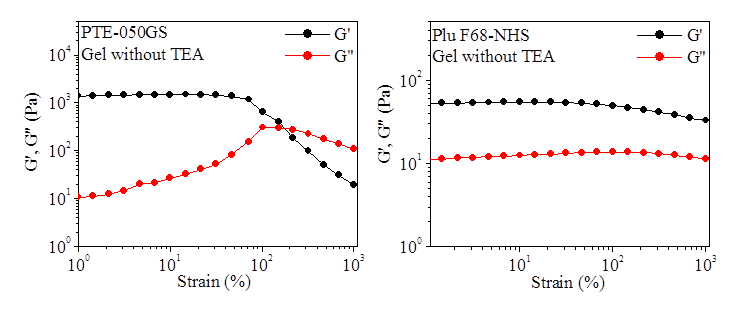


**Figure S7** Storage and loss modulus of plasma gels formed at its normal pH (7.4) in absence of TEA.


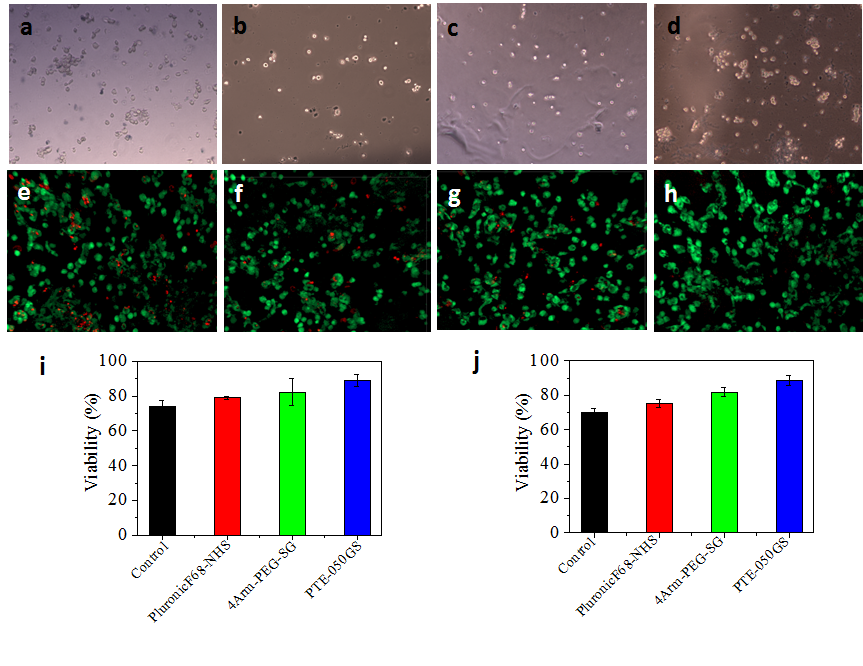


**Figure S8** Cytotoxicity analysis of the crosslinkers using (a-d) Trypan blue method and (e-h) live-dead assay method. Image (a) and (e) Control plasma gel, (b) and (f) plasma gel by 4-Arm PEG-SG, (c) and (g) plasma gel by PTE-050GS and (d) and (h) plasma gel by PluF68-NHS. (i) Quantification data for trypan blue method. (j) Quantification data for live-dead assay method.

**
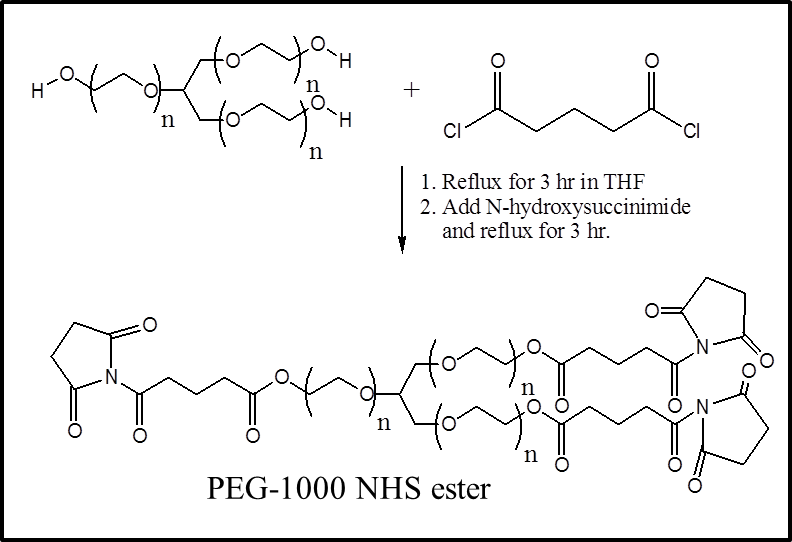
**

**Figure S9** Reaction synthetic scheme of PEG-1000 NHS ester.
